# Chrysanthemum sporopollenin: A novel vaccine delivery system for nasal mucosal immunity

**DOI:** 10.3389/fimmu.2023.1132129

**Published:** 2023-02-09

**Authors:** Jun Liu, Xiao-Dan Yan, Xian-Qiang Li, Yu-Hao Du, Li-Li Zhu, Tian-Tian Ye, Ze-Ying Cao, Zhe-Wen Dong, Shu-Tao Li, Xue Xu, Wei Bai, Dan Li, Ji-Wen Zhang, Shu-Jun Wang, Shan-Hu Li, Jin Sun, Xian-Zhen Yin

**Affiliations:** ^1^ Pharmacy laboratory, Department of Pharmacy, Shenyang Pharmaceutical University, Shenyang, Liaoning, China; ^2^ Ji-Wen Zhang laboratory, Center for Drug Delivery System, Shanghai Institute of Materia Medica, Chinese Academy of Sciences, Pudong New Area, Shanghai, China; ^3^ Department of Cell Engineering, Beijing Institute of Biotechnology, Fengtai, Beijing, China; ^4^ Lingang Laboratory, Shanghai, China

**Keywords:** nasal mucosal immunity, vaccine delivery system, chrysanthemum, sporopollenin, adhesion

## Abstract

**Objective:**

Mucosal immunization was an effective defender against pathogens. Nasal vaccines could activate both systemic and mucosal immunity to trigger protective immune responses. However, due to the weak immunogenicity of nasal vaccines and the lack of appropriate antigen carriers, very few nasal vaccines have been clinically approved for human use, which was a major barrier to the development of nasal vaccines. Plant-derived adjuvants are promising candidates for vaccine delivery systems due to their relatively safe immunogenic properties. In particular, the distinctive structure of pollen was beneficial to the stability and retention of antigen in the nasal mucosa.

**Methods:**

Herein, a novel wild-type chrysanthemum sporopollenin vaccine delivery system loaded with a w/o/w emulsion containing squalane and protein antigen was fabricated. The unique internal cavities and the rigid external walls within the sporopollenin skeleton construction could preserve and stabilize the inner proteins. The external morphological characteristics were suitable for nasal mucosal administration with high adhesion and retention.

**Results:**

Secretory IgA antibodies in the nasal mucosa can be induced by the w/o/w emulsion with the chrysanthemum sporopollenin vaccine delivery system. Moreover, the nasal adjuvants produce a stronger humoral response (IgA and IgG) compared to squalene emulsion adjuvant. Mucosal adjuvant benefited primarily from prolongation of antigens in the nasal cavity, improvement of antigen penetration in the submucosa and promotion of CD8+ T cells in spleen.

**Disccusion:**

Based on effective delivering both the adjuvant and the antigen, the increase of protein antigen stability and the realization of mucosal retention, the chrysanthemum sporopollenin vaccine delivery system has the potential to be a promising adjuvant platform. This work provide a novel idea for the fabrication of protein-mucosal delivery vaccine.

## Introduction

Nasal mucosal immunity was a front-line of immune defense to the viral invasion. Numerous respiratory viruses including severe acute respiratory syndrome coronavirus 2 (SARS CoV-2), respiratory syncytial virus (RSV) and influenza virus attack the host mainly through the mucosal route (1, 2). Therefore, the fabrication of nasal mucosal-vaccines against viral infections at the mucosal level was crucial (3). Compared to conventional systemically delivered vaccines, nasal mucosal vaccines showed great potential of safety and efficacy. Vaccines of the nasal mucosa stimulate mucosal immune responses in the lymphoid-associated tissues within the nasal region and induce the secretion of sIgA antibodies, which plays an essential role in the prevention of respiratory tract infections (4–6). Moreover, nasal mucosal vaccines also elicit systemic humoral and cellular immunity, providing more comprehensive protection (7, 8). For example, intranasal delivery of whole inactivated influenza virus vaccine induces serum IgA and IgG responses in nasal mucus of healthy adults (9). Intranasal inhalation of inactivated B. pertussis vaccine induces serum IgA and IgG and Th1 cell immune response (10, 11). Therefore, protein-based nasal spray vaccine was currently a hot research topic in the anti-viral field (12–14). However, these vaccines were limited in their effectiveness *in vivo* due to their poor immunogenicity (15). There was an urgent need to enhance the efficacy of vaccines. The development of adjuvants to improve their immune effect is one of the most important method. Although nasal mucosal vaccines could tolerate acidic solution and digestive enzymes (16), their therapeutic efficacy remained limited. The main cause is the presence of a mucosal barrier, where the nasal mucosa dilutes and traps antigens, leading to the phenomenon of antigen malabsorption (4, 17). Therefore, the administration of the mucosal vaccine requires protection against the physical elimination of antigens in the nasal cavity. It was also urgently needed to increase the stability and retention of the nasal mucosa vaccine to produce better performance.

Pollen was the male organ of flowering plants as well as the germ cell in the pistil. The hollow sporopollenin shell could be obtained by processing the pollen and removing the proteins, genetic material, pectin, cellulose and other substances. The main character of sporopollenin skeleton was a sphere-like microcapsule with a hollow structure, and the surface was either distributed with regular spines or smooth and flat or with a large number of depressions, which has better physical and chemical properties. The surface of sporopollenin has sprouting pores or sprouting grooves, which could link the inner hollow structure and the outer space. Sporopollenin was mainly composed of carbon, hydrogen and oxygen. Serval researches have shown that sporopollenin was composed of cucurbitacin, lignin, phenylpropanoid and other substances, and was a class of copolymers with large molecular weight. Sporopollenin was chemically stable and resistant to harsh chemical environments such as organic solvents, acids and bases. It also has better thermal stability (18).

In recent years, sporopollenin (Spo) has been paid close attention by experts in many fields. it’s the hollow structure was well suited for drug encapsulation. Studies have been conducted using sporopollenin for encapsulated delivery of protein-based drugs and other drugs susceptible to gastric acid breakdown (19). In addition, there were many literatures on the use of sporopollenin in oral vaccines (20). In addition, sporopollenin has been reported to be used for the adsorption of industrial oils and fats, organic wastes, and organic pesticide residues (21–23). Therefore, the unique internal cavity structure of sporopollenin was suitable as a protein drug carrier, and its outer shell could protect and stabilize the drug (24). The external morphology and structural characteristics were also preferable for nasal mucosal drug delivery by greatly increasing the adhesion and retention properties.

In this study, we designed a wild chrysanthemum sporopollenin as an adjuvant and carrier of vaccine. Squalene (Squ) and protein antigen (OVA) could be loaded on the wild chrysanthemum sporopollenin as a w/o/w emulsion, and the short spine structure on its surface could increase its retention in the nasal mucosa. The structure finally promoted the adjuvant, antigen delivery and increase the stability and retention of protein antigen.

## Materials and methods

### Reagents

Chrysanthemum powder (Taikang, Tai’an, Shandong, China); ovalbumin (Absin, Shanghai, China); squalane, Tween-80, Span-85 (Nanjing Weil Pharmaceutical, China); IgA, IgG antibodies (Abcam Multisciences, Hangzhou, China); CD4^+^, CD8^+^ (MultiSciences, Hangzhou, China); Tyrisin, PBS and penicillin-streptomycin (P-S) solutions (Hao Yang Biotechnology Co Ltd, Tianjin, China). Fetal bovine serum (FBS, extra grade, Lonza Scientific, Swiss). Anhydrous ethanol, acetone, concentrated hydrochloric acid (Fuyu Fine Chemical Co., Ltd., Tianjin, China); sodium hydroxide (Hengxing Chemical Reagent Manufacturing Co., Ltd., Tianjin, China); potassium hydroxide (Yongda Chemical Reagent Co., Ltd., Tianjin, China) phosphoric acid (Comio Chemical Reagent Co., Ltd., Tianjin, China), other chemicals and reagents not specified in the text belong to Analytical grade.

### Animals

Female Balb/c mice (6-8 weeks) and male Japanese white rabbits (3 months) without specific pathogens were obtained from the Animal Center of Shenyang Pharmaceutical University. Animals were fed ad libitum twice daily on basal chow and water free of any drugs or contaminants for 24 hours.

### Ethics statement

The procedures for feeding and testing animals shall be governed by the Regulations on the Administration of Experimental Animals approved by The State Council of the People’s Republic of China. All animal tests in this study were approved by the Animal Ethics and Experimentation Committee of Shenyang Pharmaceutical University (SYXK2018-0009). Trained and skilled animal care personnel were involved to reduce animal suffering as much as possible.

### Preparation of sporopollenin

Chrysanthemum pollen was dispersed in purified water after removing the impurities. The dispersed phase was washed repeatedly by water, 50% ethanol, 75% ethanol and anhydrous ethanol in turn until the filtrate was basically colorless. Defatted pollen was then obtained. Crude sporopollenin could be collected by filter the 85% phosphoric acid aqueous solution suspended with the defatted pollen after heat reflux with mild stirring. To obtain the dried sporopollenin refined product, crude sporopollenin was firstly washed by the following steps in order: water, NaOH (2 M), hot acetone, hydrochloric acid (2 M), acetone, ethanol and water, until the solution was colorless at each time. Finally, the refined product was collected by filtration and desiccation.

### Preparation of Spo-Squ/OVA

OVA was dissolved in water to make OVA solution, the Squ/OVA emulsion (w/o) was obtained by the mixture of OVA solution and squalene at ration of 1:1 by volume and emulsified with 8% Span-85 as emulsifier followed by sonication for 5 min. The Squ/OVA emulsion (w/o/w) was obtained by adding 8% Tween-80 and water for injection into Squ/OVA emulsion (w/o) followed by emulsify for 5 min. The Spo-Squ/OVA was obtained by the mixture of Squ/OVA and sporopollenin ultrasonically followed by addition of water for injection and dilution. The concentration of each group above was 1 mg/mL for OVA, 40 mg/mL for squalene and 20 mg/mL for sporopollenin.

### Structural characterization of sporopollenin and Spo-Squ/OVA

#### Confocal fluorescence microscopy

An appropriate amount of sample was applied to the microscope slide and covered with a coverslip. Imaging was performed by laser scanning confocal microscope (LSM 710 NLO, Germany) in sequence using laser excitation channel at wavelength of 460 nm.

#### Scanning electron microscopy

The pollen and sporopollenin samples were evenly adhered to the conductive adhesive, and the prepared samples were placed on the spraying table for 3 min for gold spraying. SEM (Nova NanoSEM 430) images were captured at 1000, 2000 and 5000 magnifications.

#### Optical microscope

A small amount of sporopollenin sample was suspended in distilled water and the sporopollenin was applied to a clean slide. Image acquisition was performed by observation made with a microscope (XD-202 type, Nanjing, China).

#### Particle size

Particle size correlation was performed using a high resolution laser diffraction particle analyzer (Sympatec, HELOS/KF, Germany), and the R3 lens was selected for focused background testing (dispersion conditions: oasisdry 5.5 bar feed rate 60%). Each sample was tested three times after background testing for lens.

#### Contact angle

Contact angle was analyzed by contact angle analyzer (KRUSS, DSA25, Germany) by following steps: the pollen and sporopollenin samples were placed on the sample stage, 500 μL of pure water was added to the flat sample at a time, the morphological changes of the water droplets on the sample were observed by filming and image acquisition.

#### SR-FTIR micro spectroscopy and imaging

The SSRF BL01B1 beamline station was applied to study the material distribution of pollen, defatted pollen, sporopollenin and drug-laden sporopollenin using transmission imaging mode. Phospholipids and drug pure products, respectively, and single-point scanning was performed using microscopic observation to detect 64 scans at an aperture of 5*5 μm and a resolution of 4 cm^-1^, and single spectrum scanning by synchrotron radiation-based Fourier transform infrared (SR-FTIR, Shanghai Synchrotron Radiation Facility) to obtain the characteristic absorption peaks of each substance. All data processing was performed using OMSNIC software. The absorption peaks of each substance were compared to determine the comparison of substances on the surface of each sample.

### Elemental analysis

Elemental analysis was performed by varioELcube elemental analyzer (elementar Vario EL III, Elementar, Germany), appropriate samples were weighed for testing and analysis to derive N and S elemental contents. The protein level of the samples was recorded based on the measured percentage of N with a multiplication factor of 6.25. The protein removal rate of sporopollenin was calculated relative to the protein content of defatted pollen.

### Examination of intranasal retention of Spo-Squ/OVA

OVA antigen was mixed with indocyanine green and samples were prepared as described above. Mice were anesthetized by inhalation in an appropriate amount of ether gas. The fluorescence intensity in mice nasal cavity was observed under an imaging apparatus (DPM, H2000), and the change in fluorescence intensity was observed after 0.5, 1, 2 and 4 h of administration. The antigen retention was examined quantitatively by recording the change in fluorescence intensity.

### *In vitro* release study of Spo-Squ/OVA

To evaluate the release of Spo-Squ/OVA, water-soluble dye was dissolved in OVA to prepare Spo-Squ/OVA, which was added to dialysis bags and the bags was dispersed in PBS buffer (pH 7.4, 37°C, 100 rpm). The dialysate was obtained at pre-determined time intervals of 0.5, 1, 2, 3, 4 h, and the drug content was measured. All data were measured in triplicate to examine the changes in drug concentration at different times.

### Mice immunization experiments

Female Balb/c mice (6-8 weeks) were divided of 3-4 mice for each group: control group, Squ/OVA, Spo-Squ/OVA, each of the above groups was administered intranasally at a dose of 25 μL free OVA(1 μg/μL) or OVA adjuvant(1:1) for one mouse; the other Squ/OVA subcutaneously at a dose of 25 μL for one mouse. Control group was PBS administered without vaccine.

The mice were immunized twice, once every two weeks. Blood samples were obtained from the retro-orbital plexus at day 28 after immunization. The blood could stand for 1 h after the clot was fully clotted. After centrifugation (HC-2062) (5000 r/min × 10 min), serum samples were preserved at -80°C and were eventually used for investigation of IgG and IgA by ELISA. Body weight was recorded daily for 28 days, Nasal washes and mucosal sections were taken from the nasal cavity of mice on day 28. Then the mice were sacrificed by cervical dislocation, and the nasal septum, spleen and thymus samples was surgically removed on day 28.

### Investigation of antigen-specific antibody titres

The antigen-specific antibody titers of serum IgA and IgG, and nasal sIgA of immunized mice were measured by indirect ELISA method. The microtiter plate was coated with the OVA antigen and incubated overnight at 4°C. Then BSA was incubated at 37°C for 2 h. After washing with PBST, diluted samples, HRP-labeled detection antibodies were added to the plate in sequence. Subsequently, add TMB chromogenic solution and termination solution (2 M H_2_SO_4_). The absorbance at 450 nm was analyzed.

### Analysis of OVA-specific T cell response

Two weeks after the second vaccination, transfer the spleen tissue to a 35 mm sterile Petri dish and add PBS containing 1 mM EDTA to the dish. Remove connective tissue or fat from the spleen tissue. Place a 70 μm cell strainer on a 50 mL sterile conical tube and rinse the strainer with medium in advance. Grind the spleen 5 times with a flattened end section with the piston of the syringe in a gentle circular motion to fully release the splenocytes. Then, splenocytes are collected and lysed with red blood cell lysate. After erythrocyte lysis, centrifuge and remove the supernatant, and wash the splenocytes with PBS. Resuspend splenocytes with 3 mL of medium. Adjust the cell concentration to 1.5×10^6^/mL and transfer to a culture plate. Then restimulated by 5 μg/mL of OVA for 24 hours. Cells were stained with fluorescein isothiocyanate (FITC)-conjugated anti-CD4, phycoerythrin (PE)-conjugated anti-CD8. Cells then were subjected to flow cytometry (BD, FASVerse) following the manufacturer’s instructions, and analyzed with FlowJo software (BD).

### *In vivo* toxicity evaluation of Squ/OVA and irritation of nasal mucosa by carrier

Control group, Squ/OVA, and Spo-Squ/OVA, were administrated by nasal, and Squ/OVA was injected subcutaneously. Weight of mice, diarrhea, drowsiness, hunchback or ruffled fur, nasal mucosal irritation and other physiological symptoms were observed and recorded daily.

The long-term nasal mucosal toxicity was evaluated in Japanese white rabbits were. The Spo-Squ/OVA was dosed at 20 mg/kg *via* the left nostril and observed the symptoms in the period of 30 min after each dose for 7 days. The number of times of various symptoms in each rabbit was recorded, and the animals were euthanized 24 h after administration. Then, the nasal mucosa was obtained to observe whether there was congestion, edema, secretion around the nostril, crusting and other phenomena, and the nasal mucosa irritation response score and irritation intensity were evaluated according to the **Supplemental Table 1**.

### Statistical analysis

Origin 8.0 and GraphPad Prism7 software were employed to perform statistical analysis and all the data were expressed as mean ± SD of means of 3~4 independent experiments. The one-way ANOVA or double t test (double-tail) were used to determine the statistical significance of differences, which was *P < 0.05, **P < 0.01, and ***P < 0.001.

## Results

### Characterization of sporopollenin carrier

**Figure 1:**
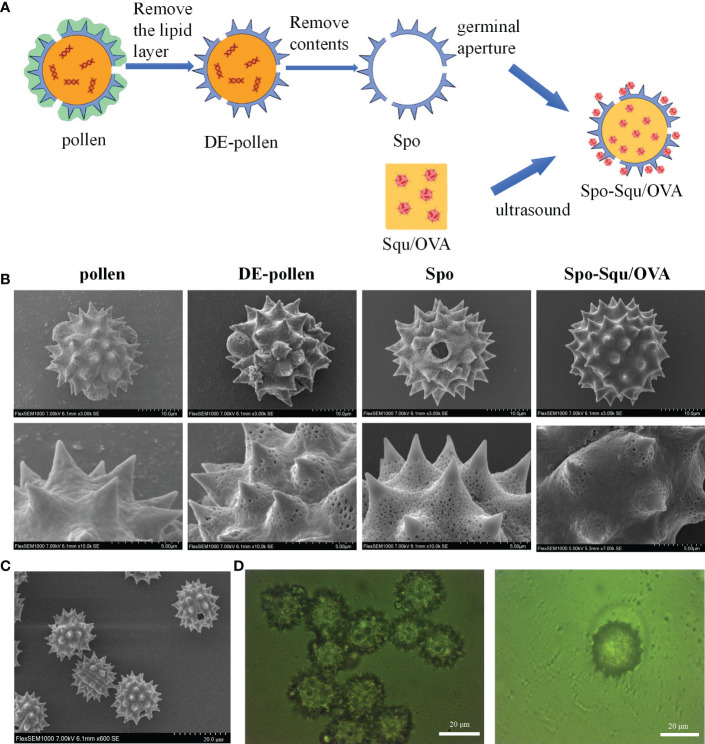
Preparation process of Spo-Squ/OVA and the shape and morphology of sporopoll en in carrie. **(A)** Schematic diagram of Spo-Squ/OVA preparation process flow chart. **(B)** SEM images of pollen, DE-pollen, Spo and Spo-Squ/OVA samples at different magnifications. **(C)** Morphology of Spo under SEM. **(D)** Morphologies under Spo-Squ/OVAfluorescence microscope. Scale bars: **(B)** 10 μm, 5 µm, **(C)** 20 μm, **(D)** 20 µm.

The pollen was spherical in shape, about 28 μm particle size, and the surface was covered with 2 μm cone-shaped spine-like protrusions, which were short and thick. Three germination pores of about 2 μm in diameter could be observed and were covered by lipid membrane in a protruding shape (**Figures 1B, C**). The outermost layer of the pollen surface was covered with lipid membrane, and the middle layer was a hard sporopollenin layer, mainly composed of aliphatic organic matter, lignin (the main source of fluorescence) (**Figure 1D**),carbohydrates and a very small amount of protein (25). The interior was protein and nucleic acid-like hereditary substance. After treatment with ethanol, a depigmented pollen(DE-pollen) was obtained, the surface of the pollen began to show depressions with reduced encapsulated material. The surface substance of the germinal pore was washed away, exposing the germinal pore structure. After treatment with phosphoric acid, the hollow structure of sporopollenin was obtained. As the protein on the surface of the sporopollenin germination pore is removed, the channel connecting the inside and outside is opened, and the structure of its layers become more obvious.

**Figure 2:**
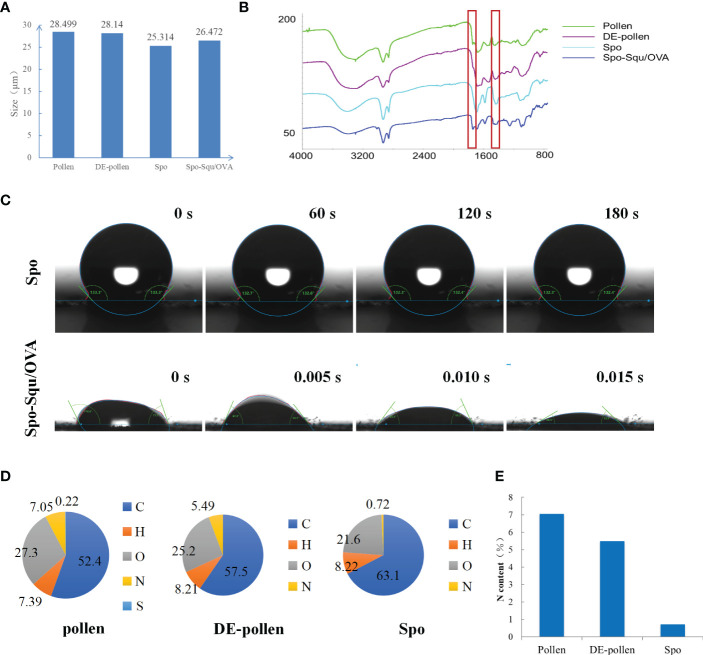
The characterization of sporopollenin carrier. **(A)** grain size patterns and **(B)** Infrared analysis of Pollen, DE-pollen, Spo and Spo-Squ/OVA. **(C)** Contact Angle diagram of Spo and Spo­ Squ/OVA. **(D)** Chemical composition analysis of pollen, DE-pollen and Spo. **(E)** The nitrogen content of pollen, DE- pollen and Spo.

The particle size of chrysanthemum pollen was 28 μm, and size of sporopollenin was about 25 μm (**Figure 2A**). These results suggest that chrysanthemum pollen and sporopollenin were more homogeneous, and the particle size was maintained after the removal of lipid film. The infrared mapping measurement results showed that the pollen infrared map had characteristic peak of phospholipid at 1600 nm **Figure 2B** (26), while the sporopollenin outer map lost the characteristic peak of phospholipid at 1600 nm, suggesting the complete removal of lipid film. As shown in **Figure 2C**, the contact Angle of Spo was 132.5° and that of Spo-Squ/OVA was 30.3°, indicating better wettability due to the presence of surfactants.

Pollen often causes allergy in the body when inhaled, and it has been pointed out that the main allergenic components were proteins and glycoproteins (27), so it was extremely important to remove the internal protein from pollen. In this study, we measured the elemental composition of pollen at each stage of treatment to affirm the dismissal of internal proteins and glycoproteins (**Figure 2D**). We focused on the content of elemental nitrogen in the pollen fraction, and elemental analysis measurements demonstrated that the content of elemental N in pollen was 7.05%, while in sporopollenin the content was 0.72%, although did not decrease to 0 due to some nitrogenous substances contained by itself (**Figure 2E**). It was suggested that the removal of protein and nucleic acid substance inside the pollen was complete. Serum IgE in mice after Spo administration was significantly lower than pollen, indicating a significant decrease in sensitization after treatment (**Supplemental Figure 1**).

### Structural characterization of Spo-Squ/OVA

Squalene was a commonly used adjuvant in a variety of vaccines (28), previous studies have shown that making w/o/w emulsion of squalene with OVA antigen exhibited better immune effects *in vivo* than adsorbing the antigen on the surface of squalene emulsion (29). It was proposed to fabricate a w/o/w emulsion of squalene and antigen and load it into sporopollenin to achieve the effect of antigen protection and antigen retardation. Meanwhile, the drug would become w/o/w emulsion under the action of two surfactants, after contacting with body fluid for release, which improved the permeability of nasal mucosa. The preparation process was shown schematically in **Figure 1A**.

After the preliminary process screening, based on the emulsification method, oil phase type and the proportion of component, we optimized the preparation conditions. Ideally, the nanoemulsion as a vaccine adjuvant with a PDI less than 0.3 and excellent size for effective uptake of epithelium on the mucosal surface as well as distribute antigen to an APC or the lymphoid tissue (17, 30). Post-optimality, squalene and Tween-80 were employed as the emulsifiers, the ultrasonic emulsification method was used to prepare the emulsion. Squalene and OVA antigen were also made into Squ/OVA emulsion.

The particle size of Squ/OVA emulsion was 354 nm, with uniform distribution of emulsion droplets. Microscopic staining to identify the results showed that the product was w/o/w emulsion.

### Enhanced nasal mucosal immune response by Spo-Squ/OVA delivery system

**Figure 3:**
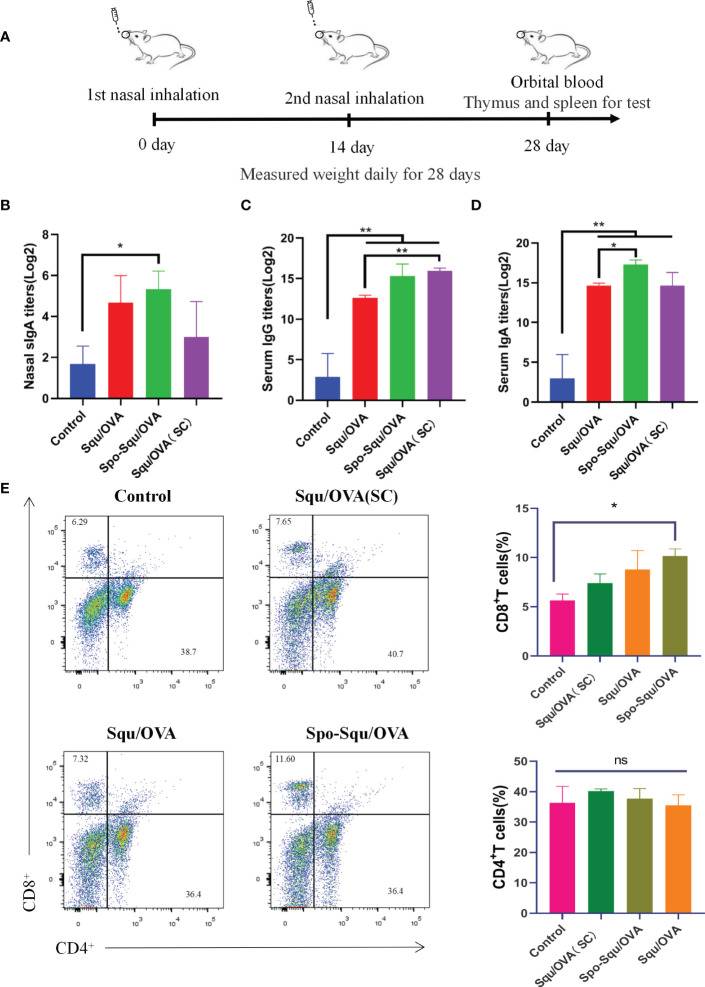
Spo-Squ /OVA enhanced systemic and mucosal immun e responses after intran asal vaccination. BALB/c mice were immunized intranasally wi th 25 µg of OVA or mixed with 25 µg of adjuvant and boosted 2 weeks later with the same formulations. Control groups re present samples coll ected from mice immuni zed with PBS witho ut antigens. **(A)** Timeline representation of experimental porceduures. **(B)** Mucosal slgA titers in nasal wash. **(C)** IgG and **(D)** IgA titers in serum. **(E)** The percentage of CD8+ T and CD4+ T cells in the spleens after ex vivo stimulation with the OVA (5 µg/ mL) for 24 hours. Significant differences were determined by double-tail t-test. Values were repotied as mean ± SEM (n=3). The asterisks indicate significant differences ("*" P<0.05, "**" P<0.01, "ns" P>0.05).

To evaluate the nasal immune adjuvant effect of Spo-Squ/OVA, two intranasal immunizations (IN) were performed in mice using PBS (control), Squ/OVA, Spo-Squ/OVA, the other Squ/OVA group is administered subcutaneously (**Figure 3A**). Local secretion of antigen-specific secreted IgA (sIgA) antibodies is a critical feature of the mucosal adaptive immunity, which plays the most significant role in preventing pathogens and toxins from adhering to or infecting epithelial cells and damaging the mucosal barrier (8, 31). As shown in **Figure 3B**, antigen immunization in the Spo-Squ/OVA induced nasal secretion of specific antibodies at a titer 3 times greater than that of control group, higher than the Squ/OVAand injection of Squ/OVA. Low IgA titers were produced when immune antigens were injected subcutaneously.

Antigens that were immunized *via* the nasal cavity not only can activate B cells, but also enter the circulation through blood or lymph in the upper cortex to trigger a systemic immune response (32). In this study, antigen-specific IgG antibodies were measured in serum to evaluate the effectiveness of the Spo-Squ/OVA as a nasal adjuvant on the systemic humoral immunity. As a result, the antigen immune-induced specific antibody IgG in the Spo-Squ/OVA had titers 5 times higher than control group, higher than the Squ/OVA and as same as injection of Squ/OVA. **Figure 3D** suggested that intranasal immunity effectively induced humoral responses. In conclusion, sporopollenin as an intranasal adjuvant and a carrier had a good effect in enhancing mucosal immunity and humoral immune response.

The antibody responses were evaluated systemically by intranasal soluble protein immunization. As shown in **Figure 3C**, intranasal immunization with Spo-Squ/OVA, induced very high serum IgA responses. The results indicated that the antigen-induced specific IgA antibody in the Spo-Squ/OVA, with titers that was 5 times higher than values in the control group, higher than the Squ/OVA (intranasal, subcutaneous) vaccination, suggesting that the mucosal immune pathway also elicited strong IgA generation.

To assess the antigen-specific T cell response ability of sporopollenin, CD4^+^ and CD8^+^ T cell responses were researched after immunization with OVA. As shown in **Figure 3E**, we used squalene as a control because it has passed clinical trials and is considered safe. The vaccine was administered subcutaneously or intranasally. Two weeks after the boost immunization, the T-cell response was analyzed using the flow cytometer (FCM) of splenocytes and re-stimulated with vaccine antigen. As **Figure 3E** shown, the highest CD8^+^ T cell responses were observed in Spo/OVA and Spo-Squ/OVA after intranasal immunization. In contrast, Squ/OVA (SC) cannot enhance the CD8^+^ T cell response. There was no significant difference in the response of CD4^+^ T cells induced by the adjuvant. Thus, Spo/OVA and Spo-Squ/OVA intranasal immunization were better CD8^+^ T cell inducing adjuvants compared to Squ/OVA (SC).

### The Spo-Squ/OVA increased nasal mucosal wettability and permeability, prolonged the retention time of antigen in the nasal cavity, and delayed the release of antigen

The Spo-Squ/OVA showed promising immunoadjuvant efficacy in eliciting systemic as well as nasal mucosal immunity. We hypothesize that its enhanced IgA production was because of prolonging residence time, delaying antigen release and increasing antigen permeability, thus providing continuous stimulation of APCs, resulting in strong activation of the IgA-secreting B cells and systemic immune responses. In order to prove this conjecture, we observed the retention time and permeability of Squ/OVA, and Spo-Squ/OVA in the nasal cavity, as well as determined their nasal mucosal wettability and released profiles, and compared the different carriers.

The result above had shown that sporopollenin was highly lipophilic with aqueous medium contact angle, oil medium contact angle, and previous studies have shown that it was a good lipophilic agent (33). In this study, the lipophilicity of Spo enabled them to be used as high-capacity carriers for loading drugs. However, the poor water wettability of sporopollenin affects its affinity and permeability of the nasal mucosa. In this study, based on a large number of experiments, we optimized the Tween-80 complex surfactant coating on the outside of sporopollenin, aqueous medium contact angle, oil medium contact angle. It was suggested that Spo-Squ/OVA could overcome the deficiency of poor wettability of sporopollenin, increase the wettability of the system nasal mucosa administration, and effectively prevent the clearance of sporopollenin by nasal mucosa.

**Figure 4:**
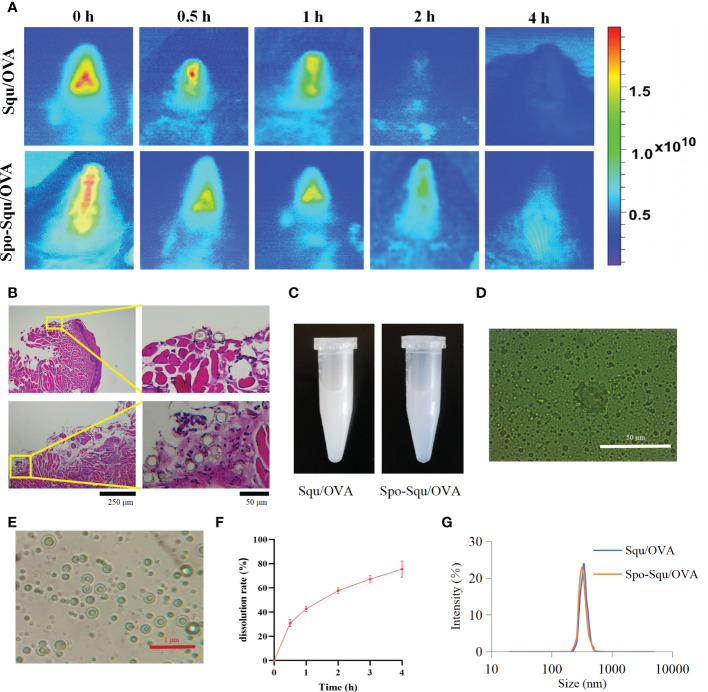
Sporopollenin increase the retention and release of antigen. **(A)** Retention of Squ/OVA and Spo-Squ/OVA in nasal mucosa. **(B)** Spo-Squ /OVA enters the nasal mucosa 4 hours after administration. **(C)** The appearance of the Squ /OVA and Spo-Squ/OVA w/o/w emulsion. **(D)** The fluorescence microscopy images of Spo-Squ/OVA. Scale bar: 50 µm. **(E)** The microscopy images of Spo-Squ /OVA. Scale bar: 1 µm. **(F)** In vitro release curve of OVA from Spo-Squ/OVA in phosphate­ buffered saline. **(G)** The particle size of Squ/OVA and Spo-Squ/OVA.

In this study, indocyanine green was applied as fluorescent dye to illustrated the retention of antigen in mice nasal cavity. As supervised by an *in vivo* imaging system, the fluorescence intensity was decreased over time after immunization. As shown in **Figure 4A**, about 85% of PBS immunization of antigen was eliminated from the nasal cavity after 1 h and almost no fluorescent signal could be observed in the nasal cavity after 2 h. The retention of Squ/OVA into the cavum nasi was improved with a lower degree than that of Spo-Squ/OVA, where the antigen retained into the cavum nasi even after immunization for 4 h. We could reasonably infer that sporopollenin plays a critical role in prolonging the retention of antigen in the nasal cavity (**Figure 4B**).

Meanwhile, the drug was released upon contact with the nasal mucosa to generate a w/o/w emulsion (**Figure 4C**). The particle size results showed that the particle size of **Figures 4D, E** was 361 nm, and the emulsion droplets were uniformly distributed.

To assess the release of Spo-Squ/OVA, the release of OVA antigen was examined by adding Spo-Squ/OVA a dialysis bag (20000 D). The release profile was shown in **Figure 4F** and Spo-Squ/OVA revealed a slower release. The particle size measurements at different times showed that the particle size remained constant in the range of 300 nm (**Figure 4G**). The effect of complex emulsion formation on antigen retention further protracted the retention time of antigen, while the strong affinity of the complex emulsion with the nasal mucosa also increased its permeability in the nasal mucosa.

### Nasal mucosal irritation and *in vivo* toxicity evaluation

**Figure 5:**
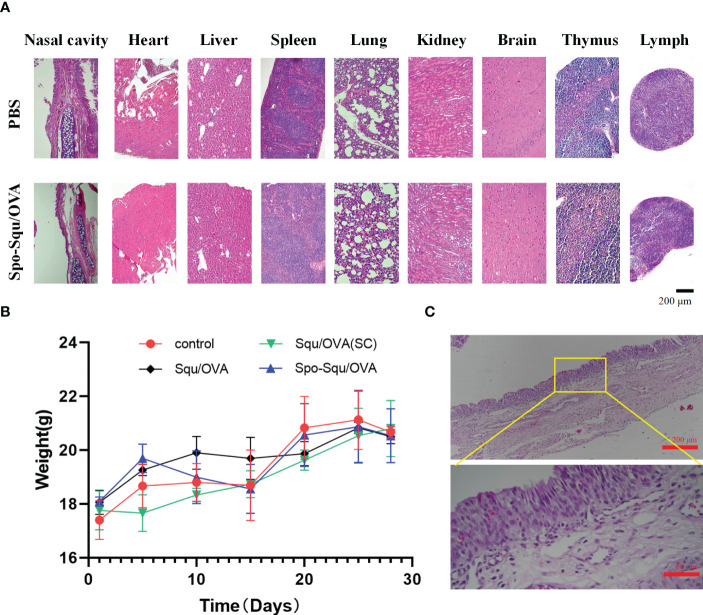
Biosafety of Spo-Squ/OVA in v ivo. **(A)** H&E staining and hi stopathological exam i nati on of different organs on day 28 after primary immunization. Scale bar: 200 µm **(B)** Body weight of mice during vaccination. **(C)** H&E staining and histopathological examination of nasal mucosa in rabbits after 7 days of continuous administration of Spo-Squ/OVA. Scale bar: 200 µm, 50 µm.

*In vivo* toxicity evaluation of nasal mucosal vaccine and nasal mucosal irritation of the vector was an important issue. In this study, the *in vivo* toxicity was evaluated administered intranasally in control group, Squ/OVA, Spo-Squ/OVA and Squ/OVA subcutaneous injection control. As shown in **Figure 5B**, mice immunized intranasally or subcutaneously had similar body weights to other mice, with stable weight gain within 28 days of immunization. No physiological signs including diarrhea, lethargy, hunchback or matted hair could be observed in each group either.

In addition, nasal mucosal irritation was evaluated after nasal administration of Spo-Squ/OVA. By comparing the HE images of the nasal mucosa of the two groups of mice, no irritation was observed in the nasal mucosal tissue compared to the blank group **Figure 5A**. By comparing the HE images of the nasal mucous membranes, no pathological characteristics caused by irritation could be observed in the nasal mucous tissue when compared to the control group.

To further investigate the long-term nasal mucosal toxicity of sporopollenin, rabbits were dosed by pipette through the left nostril 10 mg/kg of response drug for 7 days. The number of times of various symptoms in each rabbit was recorded, and the animals were euthanized 24 h after all the administration, and the nasal mucosa was dissected and removed to observe whether there was congestion, edema, secretion around the nostril, crusting and other phenomena, and the nasal mucosa irritation response score and irritation intensity were evaluated. The results indicated that this product was almost non-irritating (**Figure 5C**).

## Discussion

Most pathogens penetrate mucosal surfaces to invade the human body, Nasal mucosa was emerging as one of the most promising routes for vaccination due to its ease of accessing to mucosal tissue, elevated patient compliance and potential for self-administration (34). With the development of heat stabilized and needle-free vaccines, nasal mucosal immunization was being widely researched and developed. Needle-free nasal mucosal vaccination is suitable for mass vaccination because of its lower cost, less complicated management and waste disposal processes. Due to the rapid clearance of nasal mucosal cilia, the limited time for antigen uptake remains a large obstacle to nasal mucosal vaccine development (35). Increasing mucosal adhesion could overcome the barrier by prolonging the residence of antigen at the immune effect site (36). This article was inspired by pollen’s strong adherence to the respiratory tract due to its rough surface morphology interact with the mucosa membranes (**Figure 1B**) (37, 38).

Chemically treated pollen grains (PGs) have received much attention for their ability to be carriers (39–43). PGs were microscopic bodies containing plant male germ cells (male gametes) (44). The chemically resistant shell and the surface coating called “pollenkitt” protect the gametes from harsh environmental conditions. The sporopollenin-loaded system could achieve the potentiation effect mainly by taking advantages from the following three aspects. Firstly, sporopollenin was preferably selected as the drug carrier with pore, which facilitates drug loading and stability. The spike-like structure increases nasal mucosal adhesion and retention. Secondly, the sporopollenin drug delivery system increases the wettability of the nasal mucosal, effectively prevents the clearance of sporopollenin by the nasal mucosa, and protracts the retention time of antigen into the cavum nasi. Thirdly, after contact with nasal mucosa, the drug was released and formed w/o/w emulsion, which further prolongs the retention time of antigen. The strong affinity of w/o/w emulsion with nasal mucosa also increases the permeability in nasal mucosa.

Sporopollenins were biological microcapsules with a hollow structure and the presence of germination pores and germination grooves. The presence of many depressions on the surface could increase the specific surface. Therefore, we performed the characterization of porous materials for sporopollenin of various plants prepared previously. Chrysanthemum sporopollenin was preferably selected as a drug carrier because of its large cavity and the spiky structure. In this article, we designed a biomimetic inulin skeleton vaccine carrier. It’s a spherical structure with a diameter of about 25 μm, a short-spiked surface and an internal cavity structure connected to the outside world through three holes with a diameter of about 2 μm. The unique cavity is well suited as a drug carrier, and the sporopollenin shell was stable and protective to the drug. Squalene and protein antigen were made in w/o/w emulsion and loaded into the inulin skeleton system. Adhesion and retention experiments showed that the bionic sporopollenin drug delivery system was suitable for nasal mucosal drug delivery, with good adhesion and retention, and realized efficient delivery of nasal mucosal vaccine.

The ideal vaccine adjuvant should enhance humoral and cellular immunity and improve overall vaccine protection. After nasal vaccination, granular antigen could be cleared by the mucosal ciliary system or phagocytosed by microfolded cells in the nasopharyngeal-associated lymphoid tissue (NALT). The pathway followed by antigen in Spo-Squ should be largely like the fate of granular antigen. Antigen uptake by the APC can promote the activation of T cells and B cells. After that, the activated B cells differentiate into plasma cells that leave the follicle and secrete IgA-like antibodies into the lumen to neutralize specific antigens. Then these specific antigens could induce the secretion of serum IgA and IgG in the systemic lymphoid organs (45). Therefore, the immune response induced by agents was commonly characterized by investigating the specific sIgA and serum IgG titers as markers of local and systemic immunity, respectively (46). In this study, Spo-Squ/OVA showed higher antigen immune-inducing sIgA, IgA and IgG specific antibodies than Squ/OVA. It showed that sporopollenin as intranasal adjuvant and carrier could enhance mucosal immunity and humoral immune response. It also promoted the expression of T cell types in spleen mainly CD8^+^ T cells, thus benefitting the systemic immune response. Vaccine-elicited CD8 positive T cells may be a major mediator of protection in the early stages of the immunization (47). A study found that CD8^+^ T cells rapid and stable mobilization after prime and boost vaccination with bnt162b2, and coincides with the protective effect observed for vaccines (48). Some particulate vaccine delivery systems have been found to stimulate CD8^+^ T cell responses through the cross-presentation. In this study, we found that the sporopollenin adjuvant sufficiently induced Ag-specific CD8^+^ T cell response. Although the exact mechanism of T cells induced of sporopollenin was unclear, it appears to be superior to currently approved adjuvants that have been described to induce CD8^+^ T cell responses.

Allergies caused by substances within the pollen shell or pollenkitt are worrisome (49, 50). Therefore, these matters are ought to be dismissed prior to applying pollen shells for vaccination in order to improve safety (51). The empty pollen shells thus obtained could be filled with the vaccine of interest and the acid-resistant pollen shells could protect the vaccine from gastric degradation (52). The nasal mucosal irritation test of sporopollenin carrier illustrated its minor nasal mucosal irritation, no obvious toxicity, promising safety, and no immunogenicity of simple sporin administration.

In summary, the unique hard cavity structure of sporopollenin carrier is suitable for drug delivery and the spike structure on its surface can make its nasal mucosal retention stronger; the bionic membrane coating increases the nasal mucosal wettability; the design of w/o/w emulsion is more permeable and delays the release of antigen. This study presents a unique drug delivery system for efficient nasal mucosal delivery of protein-based vaccines, providing a new idea for the synthesis of nasal spray vaccines.

## Data availability statement

The original contributions presented in the study are included in the article/**Supplementary Material**. Further inquiries can be directed to the corresponding authors.

## Ethics statement

The procedures for feeding and testing animals shall be governed by the Regulations on the Administration of Experimental Animals approved by The State Council of the People’s Republic of China. All animal tests in this study were approved by the Animal Ethics and Experimentation Committee of Shenyang Pharmaceutical University (SYXK2018-0009).

## Author contributions

JL and X-DY contributed equally to this work. T-TY, S-JW, S-HL, X-ZY, JS and J-WZ designed experiments. JL, X-DY, X-QL, Y-HD, L-LZ and Z-YC carried out experiments. JL, Z-WD, S-TL, XX, WB and DL analyzed experimental results. JL and X-DY wrote the manuscript. All authors contributed to the article and approved the submitted version.
