# Supplementary material for: Chrysanthemum sporopollenin: A novel vaccine delivery system for nasal mucosal immunity
Source: Front Immunol. 2023 Feb 9;14:1132129. doi: 10.3389/fimmu.2023.1132129 (PMC9947463; doi:10.3389/fimmu.2023.1132129)
Supplement: Supplementary file 1 [file DataSheet_1.docx]

**Supplemental Information**

**Supplemental Table 1. Criteria for local mucosal stimulation response**

| Morphological change | Scores |
| --- | --- |
| No change or no obvious change | 0 |
| Mild hyperemia, a small amount of secretions | 1 |
| Moderate hyperemia, secretions | 2 |
| Severe hyperemia, massive secretions, edema, Mucosal degeneration | 3 |

0~0.4 was no irritation, 0.4~1.5 was mild irritation, 1.5~2.5 was moderate irritation, >2.5 was severe irritation.


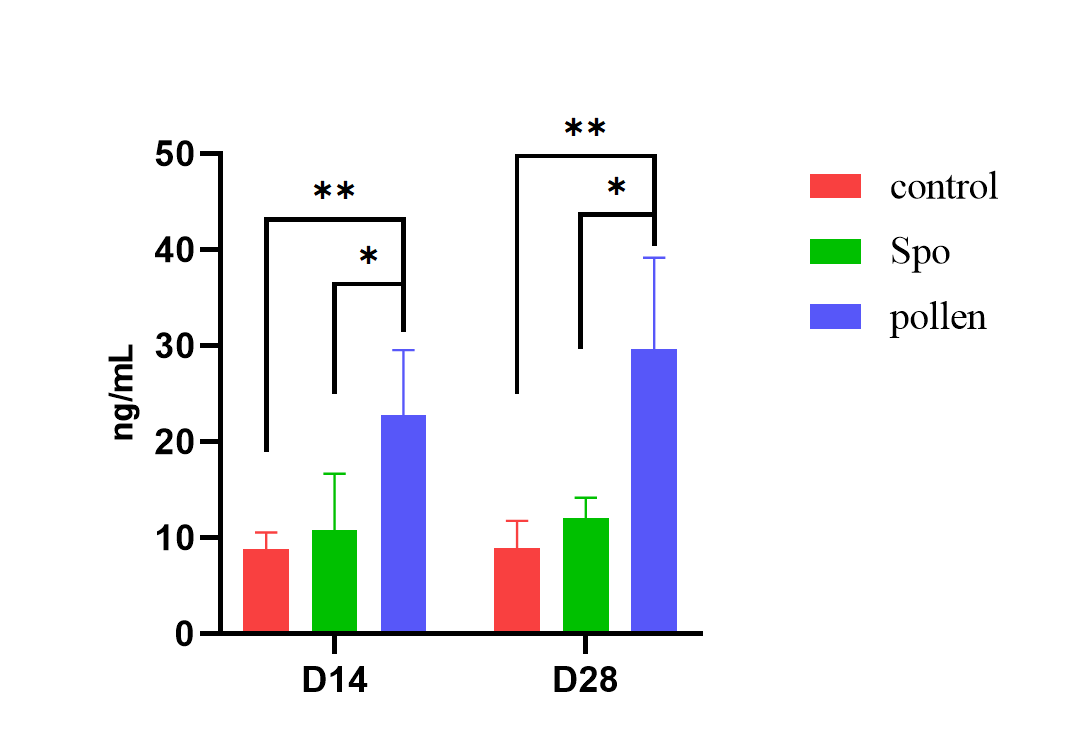


**Supplemental Figure 1.** Serum IgE antibody responses after immunotherapy with Spo or pollen in mice. Spo and pollen were administrated 2 times with two-week internals by nasal. The serum total IgE levels were measured by ELISA. n = 3, mean±SEM, *p<0.05, **p<0.01.
